# Editorial: Spectator sport and fan behavior: A prologue

**DOI:** 10.3389/fpsyg.2023.1111080

**Published:** 2023-02-01

**Authors:** Yair Galily, Ilan Tamir, Simon Pack, Ramon Spaaij

**Affiliations:** ^1^Reichman University, Herzliya, Israel; ^2^Ariel University, Ariel, Israel; ^3^St. John's University, Queens, NY, United States; ^4^Victoria University, Melbourne, VIC, Australia

**Keywords:** fans, COVID-19, sport psychology, change, soccer

[…] Life gets complicated when you love one woman but worship 11 men…

— Nick Hornby, *Fever Pitch*

In May 1987, Diego Armando Maradona led SSC (football club) Napoli to their first ever Scudetto (Lega Seria A Championship). Napoli's fanatic fans display a banner at the entrance of the local cemetery that reads “E non chano se só perso” a message to the inhabitants..... “you don't know what you missed”.

As many scholars asserted before, sport does not operate in isolation from broader society (Galily, 2019). In that respect, spectator and fan behavior in sport is vast and represents one of society's most universal leisure activities. While event attendance and media consumption of the sporting experience received a great deal of attention from scholars, there is a growing understanding that sports fans interact, both physically and digitally, with their favorite teams in numerous other ways (Dwyer et al., 2018).

Spectator sport is an expression used to portray sporting events that are attended by spectators, or fans, who watch the competition from the sidelines or stands. These sports are typically organized and governed by professional sports leagues and organizations, and they often involve teams of athletes competing against each other for a prize or championship. Spectator sports have been a popular form of entertainment and competition for centuries, with some of the earliest examples dating back to ancient Greece and Rome. In these early days, spectator sports were often associated with religious rituals and festivals, and they were a way for people to celebrate their gods and heroes.

Over time, spectator sports have evolved and become more organized and professional. Today, they are a multi-billion dollar industry, with millions of fans around the world attending games and watching them on television. Some of the most popular spectator sports include football, basketball, baseball, and soccer, among many others.

Spectator sports can provide many benefits to the athletes who participate in them, as well as to the fans who watch them. For the athletes, participating in a spectator sport can be a way to earn a living and achieve fame and recognition. It can also provide them with the opportunity to compete at a high level and push themselves to their physical and mental limits.

For the fans, spectator sports provide a form of entertainment and excitement. Watching a live game can be thrilling and exhilarating, and it can create a sense of community and belonging. Fans can also become emotionally invested in their favorite teams and athletes, cheering them on and celebrating their victories.

Indeed, as Tamir maintains in this volume, the presence of sports fans in the stands is considered a natural and essential element of most if not all sporting events. Beyond the atmosphere fans create and the color they add to the game, their presence reflects the idea that the game is more than a competition between two teams—it is a grand battle between communities and identities, which is also the reason that fans are willing to sacrifice so much on behalf of their team.

Sports have always been a source of attraction and interest and has frequently been laden with social and cultural meanings. Contrary to the business motives that characterize team owners, the relationship between the fan and the team is based on deep feelings. Sympathy exists and grows over the years from a combination of central themes: love and loyalty, community formation and building a self-identity that grows out of identification with the group (Galily, 2019; Galily et al., 2022). Actually, for the fans, there are usually two types of incentives: the first type are psychological rewards which are short-term rewards, expressed for example in feelings such as achievement, elation after a victory and excitement. The second type are social rewards which are long-term rewards resulting from the very willingness to support the team or club in any situation, these will be reflected in a sense of belonging, pride, continuity and in the strengthening of identification in the sense of “we.” At the same time, there are two additional dimensions for fanship, one is the semantic aspect of identity which contains the expressive aspect, emotional sources, the understanding they have about their sport and the ways in which they express their attitude toward their team. The second dimension, is the syntactic aspect of the identity which expresses the aggressive and competitive side, in which the fans define themselves through rivals. These two dimensions, the semantic and the syntactic, are in fact inseparable, and together, synergistically, they constituent's fan's identity (Giulianotti, 2002).

Hence, the eight papers accepted to our volume looks at fan's behavior(s) from many different angles: Min et al. investigated the interrelationships between push and pull factors associated with the consumption of women's professional basketball games; Glebova et al. Looked at the impact of digitalization on the changing face of global sports-consuming audiences, particularly from a qualitative perspective. In their article, the relationship between modern mass digital technologies (i.e., mobile applications and big data) and audiences of sports spectators was explained. Next, Jeon et al. reveal that player attractiveness and emotional experience positively affected national image and behavioral intentions (i.e., intention to visit Korea and purchase Korean products). Findings stress that foreign spectators' attitudes toward Korean women's volleyball could translate into consumption behaviors (i.e., visits and Korean products) through the national image. Subsequently, Hsiao et al. investigated the relationship between the activation of experiential marketing, satisfaction with sponsored sporting events, brand equity, and subsequent product purchase intentions in a small-scale sponsorship campaign. Correspondingly, Schut and Glebova overviewed the key points of the role of mobile applications in today's sports user experiences and test interrelations between variables related to use a smartphone on sporting events and apps' employment for sports consumers in the context of sports and a connected stadium, using an example of Roland Garros (RG) Mobile App 2018.

The on-going COVID-19 pandemic present both spectators on and off the field with various challenges side to unique opportunity to rethink the way sport fans consume and interact (Samuel et al., 2020). Consequently, the last three papers in this volume looked at distinctive fan's behaviors at times of the plague. The first looked at superstitions, which are behaviors human beings use to gain a sense of control over certain events in their lives. Thus, sport and its inherent uncertainty provide fertile ground for superstitious behavior. Research on this subject has focused mainly on athletes while examining the behavioral expressions, motivations, and characteristics of fans' superstitions that have remained marginal. However, the paper by Levental et al. aimed to address this lacuna by analyzing these behaviors as part of sports fandom and fans' daily routines. Following the outbreak of the COVID-19 pandemic, when fans' access to stadiums was restricted, their results show that the fans' absence from the stadiums led to a reduction in the quantity and frequency of their superstitious behaviors, pointing to the significance of sporting venues in fan behavior.

The second, explored football fans' experiences as they viewed “ghost games” (where teams played to empty stadiums). Tamir's paper Findings demonstrated that this unique situation, caused by the global pandemic, heightened fans' deep-rooted connection to sports and to their favorite team, and also exacerbated the social, emotional, and professional implications of viewing football games with no spectators. The last paper by Gershgoren et al., examined the perceptions of fans, athletes, coaches, and officials of the Israeli handball premier league regarding fans' contribution to the home advantage phenomenon along with the other factors (e.g., travel and officiating).

Undeniably, and as this Research Topic highlights, sporting fandom is a way of life. The agenda of the sports fan revolves around the team he is a fan of, determined according to the “league schedule” with the stadium serving as his second home. The devoted fan is obsessively engaged in searching for information about his team, attentive to the media who is involved in sports in its various forms, reads the sports sections, watches the team's games, is present on the pitches, and dressed in the colors of the club, so much so that he tends to choose the colors of his team and avoid colors associated with the opposing team. In addition, if the group is attributed a certain political affinity or ethnic affinity, this has an effect on the fan in his choice.

Last but not list, we hope that the modest contribution of this Research Topic for future research and intervention in spectators and fans management will be appreciated in the future. As Nelson Mandela once explained: […] “*Sport has the power to change the world*.” For many people around the world, it does.

## Author contributions

All authors listed have made a substantial, direct, and intellectual contribution to the work and approved it for publication.
